# Feasibility and safety of 4DryField^®^ PH as a hemostatic agent in ovarian surgery on fertility: a pilot randomized study

**DOI:** 10.1007/s00404-026-08447-7

**Published:** 2026-05-15

**Authors:** Laura Tascón Padrón, Carolin Schröder, Lucia A. Otten, Nicole Sänger, Eva-Katharina Egger, Alexander Mustea

**Affiliations:** 1 Department of Gynecology and Gynecological Oncology, University Hospital of Bonn, Venusberg Campus 1, 53127 Bonn, Germany; 2 Department of Gynecological Endocrinology and Reproductive Medicine, University Hospital of Bonn, Venusberg Campus 1, 53127 Bonn, Germany

**Keywords:** AMH, Ovarian cyst removal, Fertility, 4DryField^®^ PH, Minimal invasive fertility preservation

## Abstract

**Purpose:**

To evaluate the feasibility and safety of the hemostatic powder 4DryField® PH in laparoscopic ovarian cystectomy and its impact on fertility compared to conventional bipolar electrocautery.

**Methods:**

This is a prospective, randomized, controlled, single-center, single-blind pilot study, included in the german registry for clinical trials (DRKS-ID: DRKS00038742). The study was developed in the.

Department of Gynecology and Gynecological Oncology, University Hospital Bonn, Germany.

Twenty women (≥ 18 years old) undergoing laparoscopic ovarian cystectomy for benign ovarian lesions with baseline serum anti-Müllerian hormone (AMH) ≥ 2.0 ng/mL were enrolled and randomized equally to two hemostatic methods. Hemostasis was achieved using either bipolar electrocautery (*n* = 10) or topical 4DryField® PH powder (*n* = 10). Both groups underwent standardized laparoscopic cystectomy performed by experienced surgeons. Serum AMH, C-reactive protein (CRP), interleukin-6 (IL-6), procalcitonin (PCT), white blood cell counts, hemoglobin and hematocrit were assessed preoperatively, on postoperative day (POD) 1, and POD 14.

**Results:**

Baseline AMH levels were comparable between groups (4.54 ± 2.24 ng/mL vs. 4.39 ± 2.08 ng/mL, *p* = 0.85). By POD 14, the mean percent decline in AMH from baseline was smaller in the 4DryField group than in the electrocautery group (mean difference = 16.77%; 95% CI: -5.39 to 38.93). This difference was not statistically significant (*p* = 0.36), with an estimated effect size of *d*= 0.72 (95% CI: -0.19 to 1.62). IL-6 increased transiently on POD 1 in the 4DryField group (9.2 pg/mL [IQR 78.6] vs. 3.85 pg/mL [IQR 2.80]; *p* = 0.016) but normalized by POD 14, indicating a short, non-pathologic inflammatory response. No significant differences were observed in CRP, PCT, or leukocyte counts. No adverse events, infections, or hemorrhagic complications occurred in either group.

**Conclusion:**

In this pilot study, the use of 4DryField® PH appears feasible and safe for laparoscopic ovarian cystectomy. While the reduction in AMH levels was numerically smaller in the 4DryField group, the difference was not statistically significant. These results are hypothesis-generating and underscore the need for larger trials to evaluate the potential benefits of non-thermal hemostatic strategies for ovarian reserve preservation.

**Trial registration:**

Clinical trial; DRKS00038742.

## Take home message

**Table:** 

Use of 4DryField® PH during laparoscopic ovarian cystectomy was feasible and safe. While a trend toward a smaller decline in ovarian reserve was observed compared with bipolar coagulation, these preliminary findings suggest a potential for fertility preservation that requires confirmation in larger, adequately powered trials.	

## Introduction

Ovarian cystectomy in reproductive-age women requires a delicate balance between effective tumor removal and preservation of ovarian function. Any surgical intervention on ovarian tissue can reduce the ovarian reserve, which is crucial for fertility and endocrine function [1]. The serum Anti-Müllerian Hormone (AMH) is a reliable, cycle-independent marker of ovarian reserve [1–3]. Studies have shown that ovarian cyst removal (especially for endometriomas and other benign tumors) often leads to a postoperative drop in AMH, indicating loss of some ovarian reserve, though partial recovery may occur over time [4–6]. Therefore, preserving as much healthy ovarian tissue as possible during cystectomy is paramount in young patients desiring future fertility.

A major intraoperative challenge during ovarian cystectomy is achieving hemostasis in the cyst bed without causing unnecessary damage to ovarian tissue. Traditional hemostatic techniques like bipolar electrocautery (“bipolar coagulation”) are effective at controlling bleeding but can induce thermal injury to the adjacent ovarian cortex [7, 8]. Such thermal damage may destroy primordial follicles at the resection site and further reduce ovarian reserve [9]. This concern has driven interest in alternative hemostatic strategies that minimize collateral damage. In this context, hemostatic sealants and agents have emerged as potential tools to improve fertility-sparing outcomes. Suturing the ovarian defect can achieve bleeding control with less thermal injury, but laparoscopic suturing, is technically demanding, may prolong surgery or increase trauma, and can itself contribute to a decrease in postoperative ovarian reserve (as reflected by AMH levels) [10]. New topical agents offer a different approach as they can stop bleeding by local action and obviate the need for extensive cautery. 4DryField^®^ PH is one such innovation—a plant-derived modified polysaccharide powder that serves a dual purpose of hemostasis and adhesion prevention [11]. Upon application to a bleeding surface, 4DryField PH powder absorbs fluid and concentrates clotting factors and platelets, accelerating natural coagulation and forming a mechanical barrier to bleeding [12]. If moistened with saline, the powder transforms into a gel that coats tissues and acts as a temporary barrier preventing adhesions between raw surfaces [13].

Given these properties, 4DryField PH has been proposed as an ideal adjunct in ovarian cystectomy—it can achieve hemostasis without heat, and concurrently reduce postoperative adhesions that might otherwise impair fertility. Early case reports and pilot studies have suggested that using this agent is feasible and effective in gynecologic surgery [14]. However, rigorous evaluation of its impact on ovarian reserve and safety profile is needed to validate its role in fertility-preserving surgery.

This prospective, randomized pilot study aims to evaluate the feasibility and safety of 4DryField® PH as a non-thermal hemostatic agent during laparoscopic ovarian cystectomy, while providing preliminary, hypothesis-generating data regarding its impact on ovarian reserve as monitored via serum AMH levels. Secondary outcome was to evaluate inflammatory responses and safety profiles of the two methods.

## Material and methods

This is a prospective, randomized, controlled, single-center, single-blind pilot study at the University Hospital Bonn (Department of Gynecology and Gynecological Oncology) and it is included in the german registry for clinical trials (DRKS-ID: DRKS00038742).

The study included women scheduled for laparoscopic ovarian cystectomy for benign ovarian lesions. Patients were eligible if they were ≥ 18 years with preoperative AMH ≥ 2.0 ng/mL; exclusions included suspected malignancy or hemostatic disorders. All participants provided informed consent, and the study was approved by the institutional ethics committee (Nr.302/20). Patients were randomly assigned in a 1:1 ratio to one of two hemostasis methods: bipolar electrocautery or 4DryField PH as hemostatic agent. Randomization was conducted using simple randomization via a Microsoft Excel-generated random number list, ensuring allocation concealment by assigning treatments based on ascending patient enrollment numbers derived from the generated sequency. All patients underwent laparoscopic ovarian cystectomy performed by experienced gynecologic surgeons using a standardized technique: making a small incision in the cyst wall after identifying the cyst and carefully separating it from healthy ovarian tissue. The cysts were then removed while preserving as much ovary as possible. After cyst enucleation, hemostasis was achieved according to the randomized allocation.

In the electrocautery group (*n* = 10), the surgeon applied bipolar electrocautery to bleeding points on the ovarian wound bed until hemostasis was secured. In the 4DryField PH group (*n* = 10), the surgeon applied 4DryField PH powder directly onto the ovarian resection site for hemostasis. A thin layer of the sterile powder was insufflated to cover the entire bleeding surface. No bipolar energy was used on the ovary in this group. Hemostasis was visually confirmed at the end of surgery. Patients received standard postoperative care. All patients received a surgical drain to monitor for potential postoperative bleeding. The drain was removed on the first postoperative day (POD) and if the patients showed a satisfactory recovery, they were discharged after a clinical and sonographic gynecological examination.

Patients were followed for postoperative assessments of ovarian reserve, inflammation, and safety. The primary outcome was serum AMH level, measured at three time points: preoperatively (baseline), POD1, and POD 14. AMH was measured in ng/mL using a standardized enzyme-linked immunosorbent assay (ELISA). Secondary outcomes included inflammatory markers like C-reactive protein (CRP, mg/L), interleukin-6 (IL-6, pg/mL), procalcitonin (PCT, ng/mL) and white blood cell count (leukocytes, × 10^9/L) were measured preoperatively and on postoperative day 1 and day 14. These markers were tracked to detect any inflammatory or infectious response to the intervention. The diameter of the excised ovarian cyst (in millimeters) was recorded for each patient, either from surgical measurement or pathology report, as an indicator of cyst size. Patients were monitored for any perioperative or postoperative complications. In particular, we recorded any instances of significant hemorrhage (intraoperative or postoperative bleeding requiring intervention or transfusion), infections (fever, abscess, positive cultures), or other adverse events. Hemoglobin (g/dl) and hematocrit levels (%) were checked postoperatively to assess blood loss. Any signs of allergic or foreign-body reaction to 4DryField were also noted.

## Statistical analysis

Since this is an exploratory report of a feasibility and pilot study, we intended to inform sample size for a future trial, so no power or sample size analysis was performed a priory. Descriptive statistics were used to summarize patient characteristics and laboratory parameters. Continuous variables were expressed as means with standard deviations or Medians and IQR depending on normality of distribution. The Shapiro–Wilk test was used to assess normality. For group comparisons, independent sample t-tests were applied for normally distributed data (age), whereas the Mann–Whitney U test was used for non-normally distributed variables (AMH-, CRP- and IL6-, Leukocytes-, Hemoglobin and Hematocrit-Values). The chi-square or Fisher’s exact test was used for categorical data and comparsions are explaneid as % or OR with 95% CI. To assess longitudinal trajectories of AMH and IL-6, a mixed-design repeated-measures ANOVA was performed with time (baseline, day 1, day 14) as within-subjects factor and treatment group (4DryField vs. electrocautery) as between-subjects factor. Mauchly’s test of sphericity was significant for both AMH (*W* = 0.425, *p* = 0.001) and IL-6 (*W* = 0.006, *p* < 0.001); therefore, degrees of freedom were corrected using the Greenhouse–Geisser adjustment. The proportion of patients showing an AMH increase between day 1 and day 14 as a sign of recovery was compared between groups using Chi squared exact test. Odds ratios with 95% confidence intervals are reported as effect size measure o address potential confounding, exploratory ANCOVA analyses were performed with AMH change from baseline (day 0 to day 14) as the dependent variable, treatment group as the fixed factor, and cyst size, age, and endometriosis status as covariates. These analyses should be interpreted with caution given the limited sample size (*n* = 20).

A significance level of p < 0.05 was considered statistically significant. All statistical analyses were conducted using SPSS version 19.0 (IBM SPSS Statistics for Windows, IBM Corp., Chicago, IL, USA).

## Results

Twenty patients underwent laparoscopic ovarian cystectomy and were included in the analysis (10 in the 4DryField PH group, 10 in the electrocautery group). The two groups were similar in baseline characteristics (see Table 1). All patients had preoperative AMH values in the normal range, the mean baseline AMH was 4.54 ± 2.24 ng/mL in the 4DryField group versus 4.39 ± 2.08 ng/mL in the electrocautery group (*p* = 0.85)​.

**Table 1:** Baseline characteristics and perioperative laboratory parameters in the 4DryField® PH and electrocautery groups

	4DryField^®^ PH group (*n* = 10)	Electrocautery group (*n* = 10)	*p*-value
Age (years)	27.9 ± 4.09	24.6 ± 5.35	0.11
AMH preoperative (ng/ml)	3.96 [IQR 2.23]	4.12 [IQR 3.30]	0.85
AMH, postoperative day 1 (ng/ml)	3.27 [IQR 2.16]	3.53 [IQR 2.43]	0.96
AMH, postoperative day 14 (ng/ml)	2.93 [IQR 2.60]	2.30 [IQR 2.01]	0.36
CRP preoperative (mg/dl)	1.18 [IQR 9.45]	4.24 [IQR 8.9]	0.44
CRP, postoperative day 1 (mg/dl)	12.76 [IQR 24.3]	6.68 [IQR 3.95]	0.21
CRP postoperative day 14 (mg/dl)	2.0 [IQR 8.0]	1.55 [IQR 6.37]	0.77
IL-6 preoperative (pg/ml)	0.0 [IQR 3.75]	0.85 [IQR 2.88]	0.93
IL-6, postoperative day 1 (pg/ml)	9.2 [IQR 78.63]	3.85 [IQR 2.80]	0.01
IL-6, postoperative day 14 (pg/ml)	0.0 [IQR 2.35]	0.8 [IQR 2.33]	0.90
Leukocytes preoperative (× 10^9^/l)	6.49 [IQR 3.02]	7.34 [IQR 3.0]	0.76
Leukocytes, postoperative day 1(× 10^9^/l)	11.7 [IQR 3.54]	10.33 [IQR 3.85]	0.90
Leukocytes, postoperative day 14 (× 10^9^/l)	6.94 [IQR 2.59]	7.11 [IQR 3.21]	0.67
PCT preoperative (ng/ml)	0.03 [IQR 0.02]	0.00 [IQR 0.01]	0.35
PCT, postoperative day 1 (ng/ml)	0.03 [IQR 0.02]	0.03 [IQR 0.01]	0.56
PCT postoperative day 14 (ng/ml)	0.03 [IQR 0.01]	0.00 [IQR 0.03]	0.15
Lesion size (mm)	33.5 [IQR 26]	45.5 [IQR 31]	0.07

Note: Data are presented as mean ± standard deviation (SD) or median [interquartile range (IQR)], as appropriate

The types of ovarian cysts removed are included in Table 2. All surgeries were completed laparoscopically without need for conversion to open, and assigned hemostatic methods were used successfully in all cases. Regarding laterality, all procedures in both groups involved unilateral ovarian cysts.

**Table 2:** Histopathological diagnosis of ovarian cysts between the groups

*Histopathological Diagnosis*	4DryField^®^ PH group (*n* = 10)	Electrocautery group (*n* = 10*)*	Total (*n* = *20)*
*Endometrioma*	3	1	4
*Mature Teratoma*	4	7	11
*Mucinous Cystadenoma*	0	2	2
*Follicular Cyst*	3	0	3

**Fig. 1:**
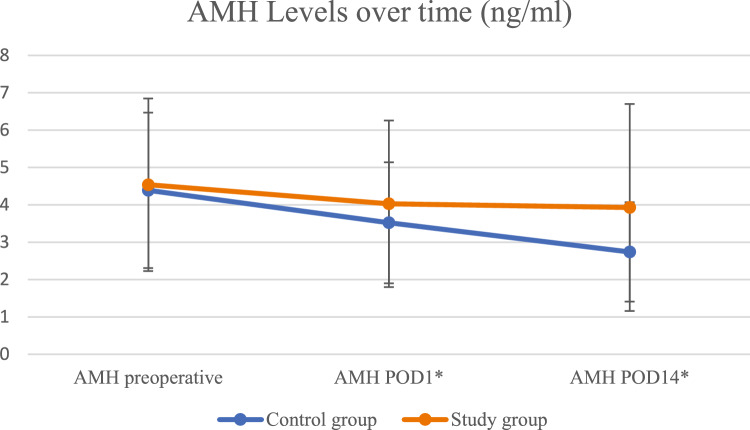
AMH levels with SD in both groups over time. *POD: Postoperative Day

Serum AMH levels over time are summarized in Fig. 1. Postoperative AMH levels declined in both groups, being it less pronounced in the 4DryField PH group. On POD1, the mean AMH was 4.03 ± 2.23 ng/mL in the 4DryField group vs. 3.52 ± 1.62 ng/mL in the electrocautery group (*p* = 0.96)​. By POD14, the 4DryField group maintained a higher mean AMH level (3.93 ± 2.87 ng/mL) compared to the electrocautery group (2.74 ± 1.33 ng/mL; mean difference = 1.18 ng/mL, 95% CI: -0.98 to 3.35, Cohen’s *d* = 0.53, 95% CI: -0.36 to 1.42). When evaluating the percent decline in AMH from baseline, the electrocautery group experienced a greater drop compared to the 4DryField group (mean difference = 16.77%; 95% CI: -5.39 to 38.93, *p* = 0.36). Although this difference did not reach statistical significance, the effect size was moderate-to-large (Cohen’s *d* = 0.72; 95% CI − 0.19 to 1.62).

**Fig. 2:**
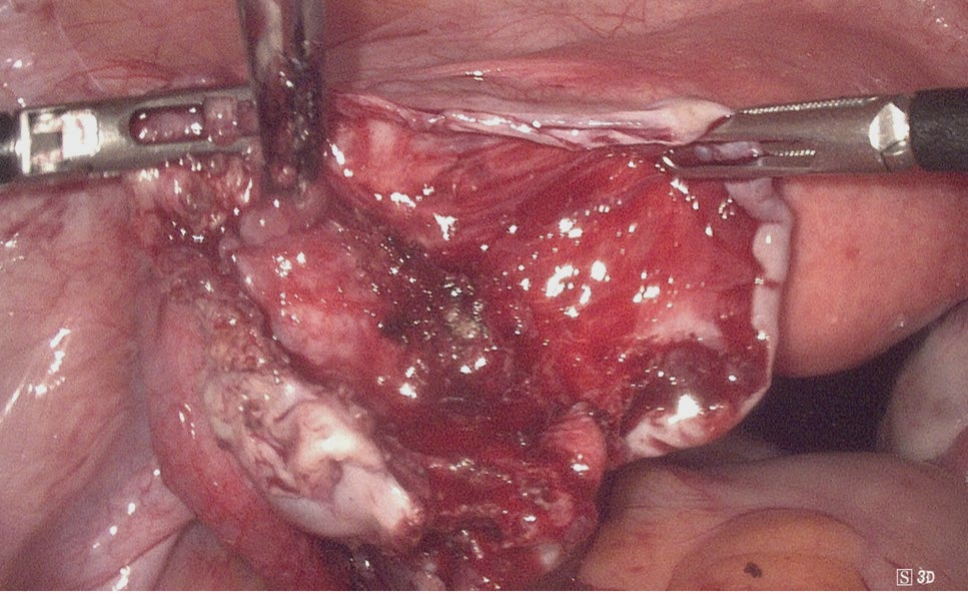
Intraoperative image after cyst removal before (Fig. 2) and after (Fig. 3) 4DryField® PH application

**Fig. 3:**
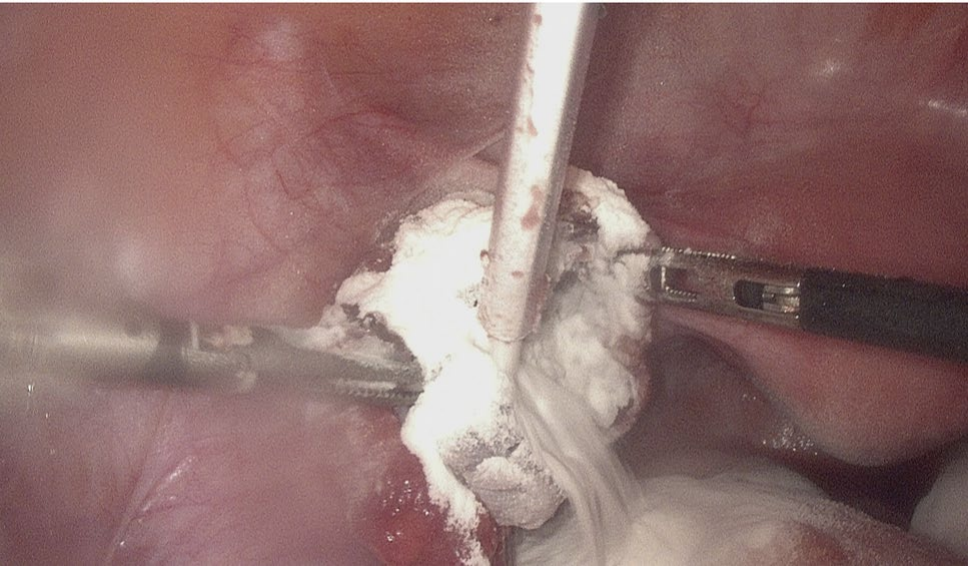
Intraoperative image after cyst removal before (Fig. 2) and after (Fig. 3) 4DryField® PH application

Assessing the amount of patients who showed an increase of AMH levels between day 1 and day 14 we observed that significant more patients in the 4DryField group showed an increment of AMH levels after surgery (70% vs 20%, *p* = 0.02, OR 9.33, 95% CI 1.2–73.0). This effect resulted less relevant after adjusting for age of the patients (Adjusted OR for 4DryField OR 11,15 (95% CI 1.01–131.75), *p* = 0.05).

Repeated-measures ANOVA revealed a significant effect of time on AMH levels (F(1.270, 22.863) = 10.646, *p* = 0.002, η^2^*p* = 0.372), indicating that AMH declined significantly across the three time points in both groups. However, the group × time interaction did not reach statistical significance (F(1.270, 22.863) = 2.244, *p* = 0.144, η^2^*p* = 0.111).

There were no significant between-group differences in CRP levels. Leukocyte counts were  similar in both groups (means ~ 11 × 10^9), PCT remained low in both groups at all timepoints.

At 24 h postoperatively, the median IL-6 concentration was significantly higher in the 4DryField group than in the electrocautery group (9.2 pg/mL [IQR 78.6] vs. 3.85 pg/mL [IQR 2.80]; median difference = 4.65 pg/mL, 95% CI: 0.60 to 67.30, *p* = 0.016). On POD14, IL-6 levels in the 4DryField group were comparable to those in the electrocautery group (0.0 pg/mL [IQR 2.35] vs. 0.8 pg/mL ([IQR 2.3] *p* = 0.90). For IL-6, repeated-measures ANOVA showed a borderline time effect (F(1.003, 18.055) = 4.358, *p* = 0.051, η^2^ *p* = 0.195) and a non-significant group × time interaction (F(1.003, 18.055) = 3.473, *p* = 0.079, η^2^*p* = 0.162). Although these results do not reach conventional significance thresholds, the effect sizes are medium-to-large (η^2^*p* = 0.162–0.195).

No statistically significant correlation was found between cyst size and postoperative AMH percent decline. Endometriomas did not have a different pattern of AMH change compared to other cyst types.

Table 3 summarizes the hematologic safety parameters, including preoperative hemoglobin and hematocrit values and their changes from baseline to POD14. No statistically significant between-group differences were found and no patient experienced clinically relevant bleeding, bleeding-related complications, or re-intervention due to bleeding.

**Table 3:** Perioperative hematologic safety outcomes in the 4DryField^®^ PH group and the electrocautery group

	4DryField^®^ PH group (median, IQR)	Electrocautery group (median, IQR)	*p*-value
*Preoperative hemoglobin (g/dl)*	13.10 [IQR 1.88]	13.15 [IQR 1.37]	0.63
*Preoperative hematocrit (%)*	37.5 [IQR 3.75]	39.0 [IQR 2.0]	0.48
*Hemoglobin change from day 0 to POD14 (Δg/dl)*	0.00 [IQR 0.98]	−0.25 [IQR 0.45]	0.14
*Hematocrit change from day 0 to POD14 (Δ%)*	0.00 [IQR 4.0]	0.00 [IQR 0.75]	0.91

## Discussion

This pilot randomized trial suggests a preliminary trend toward better preservation of the ovarian reserve with 4DryField^®^ PH compared to bipolar coagulation. However, given the small sample size and the lack of statistical significance in AMH decline differences, these observations must be interpreted with caution. Moszynski et al. reported that six months after cystectomy, patients treated with a modified starch powder had higher AMH levels and no significant AMH drop from baseline, whereas those who underwent bipolar coagulation showed a significant decline [1]. Similarly, Park et al. in a randomized trial found the AMH decline at 3 months was dramatically greater in a bipolar cautery group than in a hemostatic agent group (37% vs 13% reduction), particularly among endometriosis patients (51% vs 14% reduction) [15]. In contrast, non-endometriotic cyst cases did not show a significant difference between methods in that study, suggesting that the benefit of avoiding thermal injury is most pronounced in scenarios with inherently higher risk to ovarian reserve (e.g. endometriomas or extensive bilateral disease) [15]. Prior research often focused on endometriomas given their known impact on ovarian reserve. Endometriotic cysts are indeed associated with greater loss of ovarian reserve post-cystectomy than other benign cysts [16], likely due to both the pathological effect of endometriosis on ovarian tissue and the more difficult hemostasis often required. In our study, however, using a hemostatic powder in all cases may have attenuated such differences.

A recent meta-analysis reinforces that electrosurgical hemostasis is the most aggressive option for ovarian reserve: the mean postoperative AMH reduction is significantly greater with bipolar electrocoagulation than with suture or hemostatic sealant techniques (both in overall analyses and specifically in endometrioma cases) [17]. These findings underscore that any strategy avoiding widespread thermal coagulation can mitigate follicular loss. A 2025 phase-III randomized trial (comparing an absorbable sealant versus bipolar energy found equivalent intraoperative hemostatic efficacy, and no overall difference in postoperative hormone levels—except in the subset of bilateral cystectomies, where the sealant group had a significantly smaller AMH decline [18]. This suggests that in extensive ovarian surgery (e.g. both ovaries involved), the protective effect of hemostatic agents on ovarian reserve without compromising hemostasis may be especially clinically relevant.

By tracking early postoperative AMH changes, our study provides insight into the temporal dynamics of ovarian reserve after cystectomy with different hemostatic methods. We observed that AMH levels in the immediate postoperative period can fluctuate, dropping in the days following surgery before stabilizing or partially recovering. Literature supports this pattern: AMH often falls markedly at 1 month after ovarian cyst removal, then gradually increases by 3–6 months post-op, sometimes approaching baseline by 6–12 months [19]. In our study, the first day after surgery, both groups had some AMH decline (likely due to the inevitable loss of follicle-containing tissue during cyst excision), but the difference between groups was minimal at that point. By day 14, the gap widened, with the electrocautery group’s AMH continuing to fall. One possible explanation is that thermal damage from bipolar coagulation causes ongoing follicular atresia or impairment in the days following surgery. The heat may devascularize adjacent ovarian tissue beyond the immediate coagulation site, leading to a wave of follicle loss that unfolds over several days. In contrast, the 4DryField group, having no thermal injury, might experience only the immediate loss of follicles from the cyst excision itself, after which the remaining ovary recovers without further damage. While the electrocautery group showed a numerically larger drop between day 1 and day 14 (nearly 0.8 ng/mL), this observation did not reach statistical significance. Consequently, these findings should be viewed as preliminary evidence of a different temporal dynamic in ovarian reserve recovery. In this study we observed a transient rise in IL-6 levels postoperatively in the 4DryField group. IL-6 is a pro-inflammatory cytokine and a key mediator of the acute phase response, known to stimulate hepatic production of CRP and other reactants during tissue injury or foreign material exposure. The transient IL-6 rise reflects expected macrophage activity during biodegradation and was clinically insignificant. Ziegler et al. showed that use of 4DryField PH may induce a temporary rise in CRP post-surgery due to macrophage activity, unaccompanied by fever or elevated white cell count [11].

Postsurgical adhesions are a known cause of secondary female infertility (estimated to be involved in 20–30% of infertility cases) [20]. A randomized controlled trial of its respective application after endometriosis resection showed an 85% reduction in adhesion formation [20], resulting in a significantly higher pregnancy rate [20]. A recent review article also demonstrated that 4DryField is generally one of the most effective adhesion barriers currently available [21]. From a safety perspective, the use of 4DryField® PH was not associated with any clinically relevant differences in perioperative hematologic parameters compared with electrocautery. Moreover, no remarkable intraoperative bleeding occurred in either group, and no patient experienced bleeding-related complications or required re-intervention due to bleeding.

The strengths of this study are its prospective randomized design, and the standardized evaluation of blood samples using AMH as established marker of ovarian reserve, offering a valuable insight into the immediate endocrine impact of different hemostatic methods, an area often overlooked in prior studies Also the inclusion of variety of benign ovarian cyst types rather than focusing solely on endometriomas, make the findings more broadly applicable to clinical. The measurement of IL-6 and other inflammatory markers further adds a unique dimension, allowing an easily assessment of biocompatibility and short-term immune response to 4DryField.

However, the study also has relevant limitations. A primary limitation of this investigation is the small sample size (20 patients), which restricts the statistical power to detect significant differences in the primary endpoint of AMH decline. As the observed between-group differences do not reach statistical significance (*p* = 0.36), these findings must be interpreted as preliminary and hypothesis-generating. Secondly, the follow-up period of only 14 days captures early hormonal dynamics but does not inform long-term ovarian function, fertility outcomes, or adhesion formation. Additionally, although AMH is a well-established marker of ovarian reserve, it is not a direct measure of fertility, and other parameters such as antral follicle count or pregnancy rates were not assessed. The study was conducted at a single center by experienced surgeons, which may limit generalizability to other settings with different surgical expertise or resources. Lastly, while patients were blinded, surgeons were not, introducing the hemoglobin for performance bias despite standardized operative protocols. We observed that the 4DryField group also happened to have smaller cysts on average. This was an unintended imbalance of randomization and likely a chance finding given the small sample. It is possible, however, that surgeons might take a slightly different approach knowing the hemostatic method – for instance, if relying on cautery, one might excise more tissue to get a base that can be easily coagulated, whereas with a hemostatic powder the surgeon might favor more conservative excision of just the cyst wall, since bleeding can be managed without needing a broad base for cautery. Due to the nature of the study a relevant effect of cyst’s size ovarian reserve cannot be ruled out. Nevertheless, several studies have shown that ovarian cyst size does not significantly affect the decline in AMH levels after cystectomy [22, 23].

## Conclusion

This pilot randomized study suggests that using 4DryField^®^ PH during ovarian cystectomy may help preserve ovarian reserve, as reflected by a smaller decline in AMH levels compared to bipolar electrocautery. While differences did not reach statistical significance, the trend is consistent with previous studies and supports further investigation. Both hemostatic methods were effective and safe, and no adverse events were observed. Larger, adequately powered trials with longer follow-up are needed to confirm these preliminary findings.

## Data Availability

No datasets were generated or analysed during the current study

## Abbreviations

AMH: Anti Müllerian Hormon
ELISA: Enzyme-Linked ImmunoSorbent Assay
CRP: C-Reactive Protein
IL-6: Interleukin-6
PCT: Procalcitonin
POD: PostOperative Day
IQR: Interquartile Range
